# Role of Human Milk Bioactives on Infants' Gut and Immune Health

**DOI:** 10.3389/fimmu.2021.604080

**Published:** 2021-02-12

**Authors:** Laura E. Carr, Misty D. Virmani, Fernanda Rosa, Daniel Munblit, Katelin S. Matazel, Ahmed A. Elolimy, Laxmi Yeruva

**Affiliations:** ^1^Department of Pediatrics, University of Arkansas for Medical Sciences, Little Rock, AR, United States; ^2^Arkansas Children's Nutrition Center, Little Rock, AR, United States; ^3^Department of Pediatrics and Pediatric Infectious Diseases, Institute of Child's Health, Sechenov First Moscow State Medical University (Sechenov University), Moscow, Russia; ^4^Inflammation, Repair and Development Section, Faculty of Medicine, Imperial College London, National Heart and Lung Institute, London, United Kingdom; ^5^Research and Clinical Center for Neuropsychiatry, Moscow, Russia; ^6^Arkansas Children's Research Institute, Little Rock, AR, United States

**Keywords:** human milk, immunity, infants, neonates, development, breastmilk, immune system, gut

## Abstract

Exclusive human milk feeding of the newborn is recommended during the first 6 months of life to promote optimal health outcomes during early life and beyond. Human milk contains a variety of bioactive factors such as hormones, cytokines, leukocytes, immunoglobulins, lactoferrin, lysozyme, stem cells, human milk oligosaccharides (HMOs), microbiota, and microRNAs. Recent findings highlighted the potential importance of adding HMOs into infant formula for their roles in enhancing host defense mechanisms in neonates. Therefore, understanding the roles of human milk bioactive factors on immune function is critical to build the scientific evidence base around breastfeeding recommendations, and to enhance positive health outcomes in formula fed infants through modifications to formulas. However, there are still knowledge gaps concerning the roles of different milk components, the interactions between the different components, and the mechanisms behind health outcomes are poorly understood. This review aims to show the current knowledge about HMOs, milk microbiota, immunoglobulins, lactoferrin, and milk microRNAs (miRNAs) and how these could have similar mechanisms of regulating gut and microbiota function. It will also highlight the knowledge gaps for future research.

## Introduction

The immune system is the primary line of defense against environmental exposures such as allergens, bacteria, and viruses. The infant's immune system, often mischaracterized as “immature,” is simply naïve to its new extra-uterine environment (1). Normally it undergoes a series of pre-programmed events during early life in response to exposures that occur primarily through the respiratory tract and gastrointestinal tract (GIT) mucosa (2). The infant's immune system at birth has limited anti-oxidant and anti-inflammatory activity in the respiratory and GIT, underdeveloped physical barriers (e.g., tight junctions), limited GIT acidity (chemical barrier), delayed T-cell function and decreased secretion of immunoglobulins [specifically secretory immunoglobulin A (IgA)] (3–5). Early life in humans (from the fetal stage to early months of life) is associated with developmental milestones and human milk provides a medium for inducing both tolerances to antigens and development of a robust immune defense against harmful pathogens. Human milk feeding has been demonstrated to provide healthy GIT mucosal stimuli, impact gut microbiota composition, and promote the infant's developing immune system likely by human milk bioactives (i.e., HMOs, milk microbiota, miRNA, antibodies, lactoferrin, immunoglobulins, cytokines, and hormones) (6, 7). Careful cultivation of a healthy immune system includes not only protective responses to harmful organisms and antigens (e.g., bacteria, viruses, toxins) but moderating the response to non-harmful antigens in the environment (e.g., food antigens or beneficial commensal organisms) in the form of immune tolerance. The current review focus is on lactoferrin, immunoglobulins, HMOs, milk microbiota, and miRNAs components of human milk and their role in infants' gut microbiota colonization, gut health and immune system modulation.

## Lactoferrin

**Figure 1:**
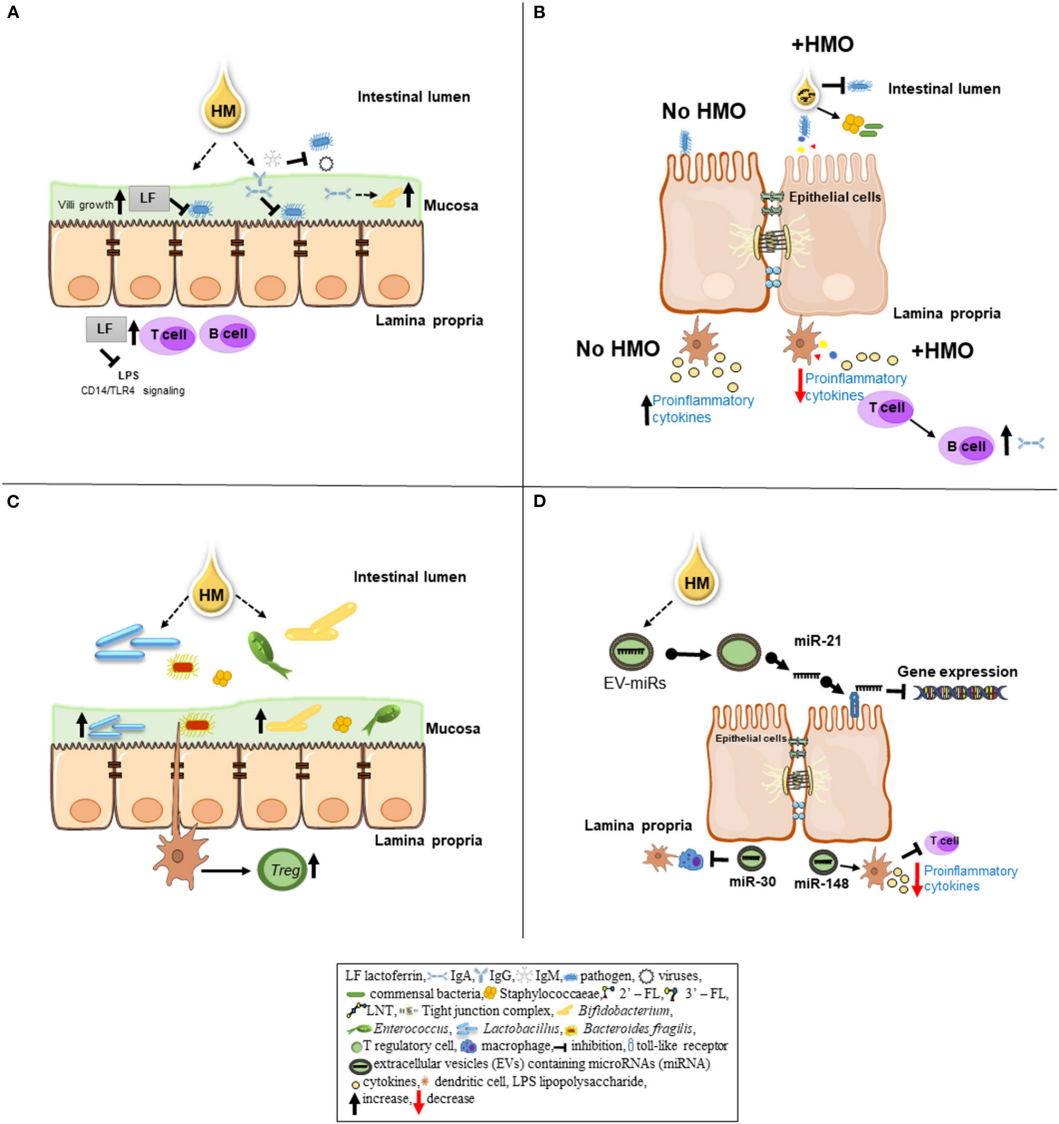
Schematic overview of specific bioactive components of human milk (HM) and their role in immunomodulation. **(A)** An iron-binding antimicrobial protein lactoferrin (LF) inhibits a number of pathogenic bacteria (i.e., *Escherichia coli*) from adhering to epithelial cell. LF can promote the growth of intestinal villi. After pathogenic bacteria invasion into the lamina propria of the epithelial gut cells, LF can inhibit the signal between lipopolysaccharide (LPS) released by gram-negative bacteria and the CD14—TLR complex (macrophage signaling). LF can enhance the maturation of B and T cells to improve the immune response. Immunoglobulins IgA, IgM, and IgG present in HM provide passive immunity to the newborn. IgA and IgG can bind to pathogenic bacteria and prevent them from adhering to the epithelial cells in the gut mucosa. Also, IgA can serve as a substrate to obligate anaerobes (i.e., *Bacteroides*) promoting a healthy microbiota colonization. IgM inhibits enteric bacterial and viral infections by opsonizing the antigen for complement fixation and destruction. **(B)** In the lumen, human milk oligosaccharides (HMO) inhibit bacterial binding to cell receptors by directly binding to the pathogens. HMOs can stimulate the growth of commensal bacteria by serving as substrates. On epithelial cells, HMOs can prevent pathogen binding by acting as binding decoy receptors. Metabolites of HMOs including short-chain fatty acids can influence epithelial cell maturation and intestinal barrier (i.e., tight junctions) function. HMOs can interact with dendritic cells present in the lamina propria leading to T-cell proliferation, subsequently, T/B cell interaction resulting in increased production of antibodies in order to keep the immune system homeostasis. In the absence of HMOs (no HMO) pathogenic bacteria binding to the epithelial cells increase cytokine production in the lamina propria as a pro-inflammatory response. **(C)** *Bifidobacterium* and L*actobacillus*, commensal bacteria found in HM, can adhere to intestinal cells, resulting in greater beneficial microbiota colonization. Furthermore, *Bacteroides fragilis* can interact with dendritic cells, resulting in suppression of inflammation by inducing T regulatory cell (Treg) production. **(D)** The extracellular vesicles (EVs) contain cargos such as microRNAs (miRNAs). EV-miRNAs likely have immunological and microbial impact on the gastrointestinal tract of neonates. Human milk miRNAs such as miR-21 can regulate gene expression by binding to toll-like receptors 7 and 8 (TLR7/TLR8). Other milk miRNAs (i.e., miR-148 and miR-30) may play a role in gut immune response by decreasing cytokine production via T-cell inhibition and preventing antigen presentation by dendritic cells and macrophages, respectively.

Lactoferrin (LF) membrane structure, membrane receptors and transport have been reviewed elsewhere (8). This section will describe the antimicrobial and immune modulatory properties of lactoferrin as well as ongoing clinical studies of formulas supplemented with lactoferrin. Lactoferrin is an iron-binding glycoprotein that exhibits immunomodulatory, anti-inflammatory, antibacterial, antifungal, and antiviral function (Figure 1A) (9–11). Human lactoferrin levels change as milk matures with colostrum having higher concentrations in both term and preterm milk (12), however, preterm milk tends to maintain higher levels of lactoferrin over time (12–14). A recent study of Chinese women reported that lactoferrin concentration was 3.16 and 1.73 g/L in colostrum and milk, respectively (15). LF binds free iron which is an essential nutrient for bacterial growth, thus leading to a bacteriostatic effect (16). Also, LF promotes the growth of low iron requiring bacteria thought to be beneficial to humans such as *Lactobacillus* and *Bifidobacterium* (17). Early studies on LF showed a fungistatic effect through iron sequestration (18, 19). Other studies have shown a more direct fungicidal interaction between lactoferrin and the fungal cell surface that is not dependent on iron (20, 21). Furthermore, *in vitro* studies in which skim human milk and bovine milk were incubated with lactoferrin, iron, and fungi (*Candida albicans*) demonstrated that skim human milk inhibits fungal growth while bovine milk did not show a fungistatic effect (22). Additionally, another *in vitro* study showed that human milk LF had higher effect in preventing bacterial growth relative to bovine LF (23) suggesting human milk LF has a superior effect over bovine milk LF. Unfortunately, not all mothers can provide breastmilk for their infants and human milk LF is difficult to obtain for research. Since human and bovine milk LF are highly similar in sequence homology and structure (24, 25), and share similar antimicrobial and immunomodulatory properties (26–29), bovine LF is used more commonly in research.

Lactoferrin has been shown to exhibit immunomodulatory properties in several animal models. For example, mice infected with *Mycobacterium tuberculosis* and supplemented with bovine lactoferrin had decreased levels of *M. tuberculosis* in their lungs as well as decreased inflammation and increased CD4^+^ and CD8^+^ cells (30). A porcine model evaluating the impact of lactoferrin on the immune system showed higher levels of natural killer (NK) cells in mesenteric lymph nodes (MLN), peripheral blood monocytes (PBMC), and in the spleen of piglets fed LF supplemented-formula compared to those fed sow milk and standard formula (31). NK cells are part of the innate immune system and provide protection to the neonate against infections as well as release cytokines that activate other immune cells (32, 33). Piglets fed formula supplemented with bovine lactoferrin had increased crypt cell proliferation and serum immunoglobulin G (IgG) compared to piglets fed formula alone (34, 35). Additionally, piglets that received bovine lactoferrin supplemented formula had greater IL-10 and TNF-α production by splenic cells when compared to the control group (35). Collectively, lactoferrin likely plays a key role in the immune response in neonates. Due to these antimicrobial and immunomodulatory properties of lactoferrin, lactoferrin supplementation in preterm infants has been attempted to decrease late-onset sepsis and necrotizing enterocolitis (36). Moreover, the antifungal property of LF is quite important as premature infants are much more susceptible to fungal infections. Thus, several studies of formulas supplemented with bovine LF to support infants' growth and development have occurred. For example, infant formulas supplemented with bovine LF at 0.6 and 1.0 g/L (range of LF concentration found in mature human milk) were compared to a standard cow's milk formula evaluating growth and tolerance in healthy term infants from 12-days old to 12 months of age. This study reported no growth rate difference between formulas, however the bovine LF supplemented formulas had softer stool consistency relative to the infants fed standard formula (37). Several studies have investigated the addition of bovine LF to neonatal diet (breastmilk, donor milk, and/or formula) in premature infants and have not found significant differences in late onset sepsis outcomes (38–40). Future studies are needed to determine the beneficial effect of enteral LF and LF addition to formulas to enhance the anti-pathogenic effects and immune response in term as well as in preterm infants.

## Immunoglobulins

Immunoglobulins (Igs) are glycoprotein molecules produced by plasma cells. They have been shown to provide passive immunity to infants via transfer across the placenta and during breastfeeding. There are five different types of Igs—IgA, IgG, IgM, IgE, and IgD; however, only IgG, crosses the placenta with the majority being transferred in the 3rd trimester (41, 42). All types of Igs have been found in human milk with the most predominant being secretory IgA (sIgA) followed by sIgG (43). sIgA protects against toxins, bacteria, and viruses by preventing binding to the host or directly neutralizing, and serves as the first line of defense in the intestines (Figure 1A) (44–46). sIgA in milk is only partially digested in the stomach of both preterm and term infants while the remainder survives to provide immunity to the lower GI tract (47). Levels in human milk decrease over the first 12 weeks post-partum, most significantly over the first week (48, 49). Although it does decrease, infants rely on human milk sIgA initially, as the cells that produce sIgA in the neonatal gut are low at birth and increase by 10–20 times over the first 6 months of life (50). This correlates with a study comparing fecal sIgA levels in breastfed and formula-fed infants which noted that in the first month of life, sIgA levels were much higher in the breastfed group but were more similar between breastfed and formula fed infants at 6 months of age (51). In mothers immunized with the *Neisseria meningococcal* vaccine, IgA antibodies specific for *Neisseria meningitidis* have been shown in human milk for up to 6 months post-partum (52). Interestingly, mother's health status appears to impact sIgA levels in the human milk. sIgA levels have been reported to be lower in the mature milk of mothers with gestational diabetes (53) and in mothers with post-partum stress, anxiety, and depression (54). IgA and IgG levels are lower in the colostrum of mothers with gestational diabetes compared to normo-glycemic women (55, 56). Overall, data suggests that mothers' health condition, vaccination status and lactation period impacts IgA levels in human milk.

IgG is the main immunoglobulin found in serum and is associated with long-term immunity. It not only activates the complement cascade to remove pathogens, but has also been shown to protect against viral infections at the mucosal level through neutralization (57, 58). IgG levels in human milk are low, but increase over time (59). Interestingly, the concentration of IgG is higher in the human milk of exclusively breastfeeding mothers compared to those that are non-exclusive breastfeeding (59). In a mouse model, pathogen-specific IgG was shown to be transferred in milk and protect the pups by coating the pathogen and reducing intestinal colonization (60). Kazimbaya et al. (61) collected human milk samples from mothers prior to their infant receiving the live rotavirus vaccine. For each sample, whole milk, purified IgA, purified IgG, and IgA/IgG depleted milk were isolated. MA104 cells inoculated with the live rotavirus vaccine were exposed to different dilutions of whole milk, purified IgA, purified IgG, and IgA/IgG depleted milk. Interestingly, whole milk and purified IgA and IgG inhibited viral replication suggesting that human milk IgA and IgG can protect against rotavirus infections (61). These studies suggest that human milk IgG plays a role in decreasing infections in infants.

IgM is also transferred to infants via human milk. IgM levels do not vary in human milk in exclusive breastfeeding mothers compared to non-exclusive breastfeeding mothers (59). However, IgM is partially digested by term infants while it is not digested by preterm infants (62). Nevertheless, IgM antibodies protect against bacterial and viral infections by opsonizing the antigen for complement fixation and destruction (63, 64). Serum IgE is associated with a reduction in allergic reactions and parasitic infections. It has also been shown to protect against viruses such as parvovirus B19 (65) and progression of human immunodeficiency virus 1 (HIV-1) (66, 67). Anti-parvovirus B19 IgE antibodies have been found in human milk (68), which might help protect breastfed infants from infection with parvovirus B19. Allergen-specific IgG and IgE antibodies are present in both maternal blood and human milk which may sensitize infants to similar allergens (69). IgD is expressed on mature B cells and it has been shown to bind to certain bacteria resulting in B cell stimulation and activation (70, 71).

Of note, IgA, sIgA, IgM, and IgG concentrations are significantly higher in fresh human milk compared to donor milk (62), which is important to infants in the neonatal intensive care unit due to frequent use of donor milk. This is not unexpected as most donor milk is from mothers of infants that are at least 6 months of age and these samples undergo Holder pasteurization. IgM and IgG are more sensitive to Holder pasteurization than IgA (62, 72, 73). Overall, Igs play a role in reducing pathogenic infections, allergies and likely gut maturation in combination with other components of human milk.

## Human Milk Oligosaccharides Promote Beneficial Microbiota Growth, Protect From Inflammation, and Prevent Pathogen Invasion

Human milk oligosaccharides (HMOs) are unconjugated lactose-based carbohydrate structures (74, 75) with concentrations between 7 and 14 g/L in mature milk and 20–24 g/L in colostrum, making HMOs the third most abundant solid component in human milk after lactose and lipids (74, 76). The milk oligosaccharide profile in human milk is more diverse than that of other mammals. For example, the concentration of oligosaccharides in bovine milk is 100 mg/L, and only 50 oligosaccharides structures have been identified in bovine milk (77). However, more than 200 distinct HMO structures have been identified in human milk (74, 75, 78, 79). The structure of HMOs has been reviewed previously (80). The HMOs profile among individual women varies due to differences in the expression of the secretor (Se) and Lewis (Le) genes in the mammary gland. The Se gene encodes for α1,2-fucosyltransferase 2 (FUT2) while the Le gene encodes α1-3/4-fucosyltransferase 3 (81, 82). A systematic review to determine the most abundant HMOs comparing both term and preterm milk reported that for secretor mothers, term milk is most abundant with the neutral HMOs 2′-fucosyllactose (2′FL), difucosyllacto-N-hexaose II (DF-LNH II), Trifucosyllacto-N-hexaose (TF-LNH), and Lacto-N-Fucopentaose I (LNFP-I) and the acidic HMOs 6′-sialyllactose (6′SL), Disialyllacto-N-Tetraose (DS-LNT), and fucosyllacto-N-neohexaose I (FS-LNnH I). For secretor mothers, preterm milk is most abundant with the neutral HMOs 2′FL, DF-LNH II, LNFP-I, and tetrasaccharides lacto-*N*-tetraose (LNT) and acidic DS-LNT, 6′SL, sialyllacto-N-tetraose c (LST c). Non-secretor milk does not contain α1-2-fucosylated HMOs (83). Additionally, this study revealed that non-secretor term milk is most abundant with neutral DF-LNH II, LNT, and lacto-*N*-neotetraose (LNnT) and acidic 6′SL. Non-secretor pre-term milk is most abundant for neutral DF-LNH II, LNT, and LNFP II and acidic DS-LNT, LSTc, and 6′SL (83). Erney et al. (84) evaluated 435 women from 10 countries and showed a significant variance in expression of HMOs. In particular, European and Latin American mothers had higher 2′FL expression than those in the US or Asia (84). An in-depth evaluation of regional variation in HMO composition evaluating 410 women from 11 different regions in Europe, North and South America, and sub-Saharan Africa showed variation in secretor status based on regions and self-identified ethnicity (85). It also noted variation in total HMO concentration as well as concentrations of all HMO types except LNFP-I. In addition, several HMO concentrations varied based on environment (rural vs. urban Gambia) including higher LNnT and DSLNT in the rural cohort (85). In addition, HMO composition is likely impacted by exercise. For example, recently Harris et al. (86) demonstrated that exercise induces an increase in 3-SL in human and mice during lactation. In conclusion, HMO composition is impacted by geographic location, likely diet, the secretor status of the mother, term vs. preterm milk and exercise. Thus, future studies need to determine how combination of these factors can optimize HMO synthesis and protect neonates during the infancy period.

### HMOs Promote Growth of Healthy Gut Microbiota and Exhibit Protection Against Infections

HMOs have been shown to have a prebiotic effect as they are not digested in the gut and reach the large intestine intact where they are utilized by gut microbiota. HMOs have been shown to stimulate gut microbiota growth and composition. *Bifidobacterium*, specifically *Bifidobacterium longum* subsp. *infantis* and its interaction with HMOs has been well-studied. *B. infantis* has greater growth when HMOs, not glucose, are the sole source of carbohydrates (87). Its genome has been shown to contain gene clusters dedicated to HMO metabolism and utilization (88). This ability to grow and metabolize HMOs is not present across all bacteria, but seen in *B. infantis* as well as *Bifidobacterium bifidum, Bacteroides fragilis*, and *Bacteroides vulgatus* (89–91). Many bacteria, *Lactobacillus gasseri* and *Enterococcus*, for example, do not grow well, or at all, in just the presence of HMOs (87, 91). In a recent animal study, healthy rats were supplemented daily with 2′-FL from days 2 to 16 of life. At day 8, supplemented animals were noted to have increased villus heights as well as higher *Lactobacillus* proportions in cecal samples. At day 16, animals had higher plasma IgA and IgG as well as more T-cell subsets in their mesenteric lymph nodes (92). This study shows that 2′FL supplementation early in life has a prebiotic effect as well as promotes intestinal growth and immune system maturation.

HMOs not only promote a healthy gut microbiota composition, but also have antimicrobial properties. For instance, α1,2-fucosylated oligosaccharides inhibited *Campylobacter jejuni* infection in mice (93). In addition, 2′FL percentage in milk has been shown to be inversely proportional to rates of *C. jejuni* diarrhea (94). HMOs have also recently been shown to have antimicrobial properties against *Streptococcus agalactiae* [Group B Strep (GBS)], *Staphylococcus aureus*, and *Acinetobacter baumannii* (95, 96) by increasing the sensitivity of such bacteria to several antibiotics, particularly antibiotics to which they are not usually susceptible (97). Overall, HMOs provide some protection to infants against bacterial pathogens.

HMOs protect infants from pathogen invasion by various mechanisms (Figure 1B). Several *in vitro* and *in vivo* studies highlighted the antiviral properties against different viruses including rotavirus, norovirus, HIV, and influenza. Rotavirus is the most common cause of severe diarrhea worldwide and accounts for 5% of all deaths among children <5 years of age (98). *In vitro*, 2′FL, 3′SL, 6′SL, and galacto-oligoasccharide reduce infectivity of human rotavirus in MA104 cells, mainly through effects on the virus (99). In experimental settings, 2′FL, LNnT, 3′SL, and 6′SL supplementation in piglets acutely infected with rotavirus downregulated the viral non-structural protein-4 (NSP-4) mRNA expression in the ileum, indicating HMOs inhibit rotavirus replication in the gut (100). Other animal studies in both rats and piglets show that HMOs, in addition to prebiotics, can reduce the length of diarrhea caused by rotavirus (101, 102). HMOs have also been shown to protect against norovirus, the most common cause of acute gastroenteritis outbreaks. Norovirus has been shown to interact with histo-blood group antigens differently with type O having higher susceptibility and B having lower susceptibly to the infection (103, 104). Non-secretors have also been shown to have lower susceptibility to norovirus infections. However, milk from non-secretor mothers does not inhibit attachment of norovirus while milk from secretors does (105). This is likely due to 2′FL binding to the virus and blocking attachment to the gastrointestinal tract (106, 107). 3′FL has also been shown to bind norovirus and block its attachment. Both 2′FL and 3′FL do so by binding to the HBGA pockets on the norovirus capsule, thus, they act as soluble decoy receptors to block pathogens (106). Human milk with higher LDFH-I levels is associated with protection against norovirus as well (94). In both of these gastrointestinal viruses, HMOs have been shown to improve outcomes.

It is estimated that over 38 million people are living with HIV and the rates of transmission from mother to child are as high as 45% (108). In the western world, HIV is considered a contraindication to breastfeeding (109), however, in other countries where access to clean water is unavailable, it is deemed to be the safest option for infant feeding due to lack of nutritional alternatives (110). While breastfeeding is the main post-natal transmission route, many breastfed infants do not become infected. HMOs have been shown to bind the HIV surface glycoprotein, gp120 and decrease binding to dendritic cells (111). HIV infected mothers, particularly those with higher concentrations of LNnT are less likely to transmit HIV to their infants. Mothers with higher concentrations of 3′SL are noted to have higher transmission rates to their offspring as well as a higher viral load and lower CD4 count (112, 113). Higher concentrations of fucosylated HMOs are also associated with decreased mortality in non-infected infants whose mothers are HIV positive (114). Another viral infection that can be ameliorated with HMOs is influenza. Influenza infects more than 3 million people yearly worldwide and causes over 300,000 deaths (115). An *in vitro* study using pretreated respiratory epithelial cells (Calu-3, 16HBE lines) and PBMCs challenged with either respiratory syncytial virus or influenza and incubated with various concentrations of 6′SL, 3′SL, 2′FL, and LNnT for 24 h showed that 6′SL and LNnT significantly decreased influenza viral load in both airway epithelial cell lines (116). In addition, modified versions of 3′SL and 6′SL have been shown to block hemagglutination and prevent infectivity of influenza viruses (117, 118). HMOs have been shown to improve outcomes in viral gastroenteritis and influenza as well as impact transmission of HIV.

### HMOs Improve Gut Barrier Function and Optimize Immune Function

Necrotizing enterocolitis (NEC), a common intestinal disease among premature infants, can cause significant morbidity and mortality [reviewed by Neu and Walker (119)], and is far less common in human milk fed vs. formula fed infants (120). Enteral feeding, including breast- and formula-feeding, impacts the gut maturation of neonates by increasing or decreasing intestinal permeability (121, 122). Decreased intestinal permeability is associated with gut maturation while elevated permeability makes neonates more susceptible to enteric infections and inflammation such as NEC (123, 124). Several studies in animals and humans demonstrated that HMOs may contribute to breastfed infants' lower rates of NEC. In a NEC induction model using neonatal mice, HMO supplemented formula-fed pups had increased mucin expression and decreased intestinal permeability (125). In another rat model of NEC, pups fed HMO supplemented formula had improved survival and the HMO disialyllacto-N-tetraose (DSLNT) was noted to be protective (126). Formulas supplemented with 2′FL have been associated with decreased NEC rates in both mice and rat models (127, 128). However, animal models using preterm pigs have shown only minor effects of HMO supplemented formula on gut microbiota (129) and no effects on gut permeability (130). In addition, several studies have found that milk with lower levels of DSLNT is associated with higher rates of NEC (113, 128). In breastfeeding or pumping mothers, decreased diversity of HMOs, specifically lower concentrations of LNDFH-I during the first month of life is associated with a higher risk for NEC development in preterm infants (131). Clinical trials reported an association of breastfeeding with decreased intestinal permeability at 7 and 14 days of life in preterm infants compared to those that were formula fed (122). In preterm infants, decreased intestinal permeability was associated with increased abundance of *Clostridium* and *Bifidobacterium* during the first 2 weeks of life (132). However, which components of human milk are providing these effects and interactions remains to be determined. Overall, HMOs have been shown to decrease pro-inflammatory cytokine expression, pathogenic bacteria penetration, and intestinal permeability in the gut (125, 133, 134). These findings suggest that not just HMOs alone, but rather HMOs in combination with maternal and/or host microbiota might regulate the intestinal barrier function.

HMOs play an important role in the enhancement of the immune system both locally and systemically. HMOs enhance the functions of human dendritic cells (135), an antigen-presenting cell that plays a pivotal role in the regulation and development of the immature immune system in neonates through the recruitment of functional regulatory T-cells (136). For instance, an *in vitro* approach showed that 0.8, 2 and 5 mg/mL of an HMO mixture upregulated interleukin production (IL-10, IL-27, and IL-6) in dendritic cells (135). Furthermore, HMOs at these concentrations protected dendritic cells against the inflammatory impact of 5 mg/mL lipopolysaccharide (LPS) (135). In a recent mouse model, neutral HMO fractions stimulated the immune response in peritoneal macrophage cells by upregulating the release of nitric oxide (NO), prostaglandin E2 (PGE2), reactive oxygen species (ROS), TNF-α and interleukins such as IL-1β, IL-2, IL-6, and IL-10 (137). Therefore, it is reasonable to hypothesize that certain HMOs can inhibit the pro-inflammatory responses in breastfed infants. In a mouse model, 2′FL supplementation with a dose range of 0.25–5% (w/w) 2 weeks before the primary and booster vaccinations enhanced humoral and cellular immune response to vaccines (138). Mice that received 2′FL had increased levels of vaccine-specific IgG1 and IgG2a in the serum that were 2′FL dose dependent and increased CD27 expression in splenic B-cells. When stimulated *ex vivo*, spleen cells from 2′FL mice had increased interferon-γ production and proliferation of CD8^+^ and CD4^+^ T-cells (138). In addition, mice that were fed the 2′FL containing food had increased activation of B-cells, T1-helper cells, and regulatory T-cells in their MLN (135). In a porcine model, piglets that received formula supplemented with HMOs were shown to have increased circulating NK cells and mesenteric lymph node memory T-cells compared to those that only received formula (139). These studies show that HMOs improve immune response to both infections and vaccines.

HMOs have been shown to play a role in toll-like receptors (TLRs) expression. TLRs are a family of pattern recognition receptors that play a key role in the recognition of invading pathogens and initiate host defense (140–142). Studies have reported structure-dependent effects of HMOs on TLR functions. For example, Asakuma et al. (143) showed that 3′SL, 6′SL, and 6′GL increased expression of both TLR2 and TLR4 while LNFP-I upregulated TLR4 in intestinal cell line HT-29 (143). In another *in vitro* study, Cheng et al. (144) reported that 3′-FL activated TLR2 whereas LNT activated several TLRs in THP1 macrophages. They also found inhibitory effects for HMOs on TLRs *in vitro*. For instance, 6′SL, 2′FL, and LNnT inhibited TLR5 and TLR7 whereas 3′FL inhibited TLR5, TLR7, and TLR8 (144). A recently published study fed mice and premature piglets with 2′FL, 6′SL or lactose supplemented formula. Those fed 2′FL and/or 6′SL were noted to have decreased signs of NEC. 2′FL and 6′FL inhibited TLR4 signaling *in vivo* in cultured IEC-6 enterocytes, in human intestinal explants from NEC patients, and in mouse derived enteroids (145). These studies indicate some role for HMOs in modulating TLRs, however, comparisons are difficult due to differences in studies conducted. The complex effects of different HMOs in modulating TLRs need to be investigated through *in vivo* models. This will enable us to determine the different mechanisms involved in immune modulation by HMOs. Overall, HMOs appear to have a protective effect in reducing inflammation and inducing stronger immune response.

### HMOs as Supplements to Boost Immune Function

HMOs and bovine milk oligosaccharides (BMOs) are currently being studied for their ability to improve immune response in infants. Bovine milk serves as a source of simple and complex oligosaccharides that resemble HMOs (146). It is substantially lower in overall total oligosaccharide concentration compared to human milk, however, there are some similarities in the oligosaccharide profile (147). Bovine milk has a much larger proportion of acidic oligosaccharides including 3′SL and 6′SL as well as neutral LNnT, which are identical to the HMOs with the same name (148). Fucosylated structures such as 2′FL have also been isolated from bovine milk, though in far lower concentrations than human milk (146, 148). BMOs have been demonstrated to elicit similar biological functions to those of HMOs including inhibition of pathogen adhesion to intestinal enterocytes, diminished gut permeability, decreased inflammatory markers, and correction of gut dysbiosis (149). Charbonneau et al. (150) investigated breastfed infants' growth parameters and differences in human milk oligosaccharide composition in Malawi (150). This study demonstrated that the human milk of mothers whose infants had poor growth had lower levels of sialylated HMOs and overall lower concentrations of HMOs (150). Based on this data, a germ-free mouse and piglet model was then used to investigate the impact of sialylated HMOs on stunting phenotype. Animals were gavaged with bacterial strains from feces of infants with growth failure and fed a typical Malawian diet. Some of the animals were supplemented with sialylated BMO's (S-BMO) as well. Those that received S-BMO had improved lean body mass gains, improved metabolism, and elevated levels of N-acetylneuraminic acid (150), suggesting sialylated oligosaccharides are involved in infant growth.

Addition of synthesized oligosaccharides to infant formulas is an evolving field. 2′FL is one of the most abundant and well-studied of the human oligosaccharides as previously mentioned. It has been successfully synthesized and shown to be structurally similar to 2′FL found in human milk samples (151). In a neonatal piglet model, enzymatically synthetized 3′SL and 6′SL sodium salt supplemented bovine based formulas were investigated (152, 153). Piglets were fed either a control diet or concentrations of 140, 200 or 500 mg/L 3′SL, and 300, 600, and 1,200 mg/L for 6′SL. These studies showed that the synthesized HMOs are safe and maintain similar growth in supplemented piglets compared to control diet (152, 153). Several clinical studies have evaluated the addition of 2′FL to formula. 2′FL formula fed infants were compared to breastfed infants and all infants had appropriate growth (154). An evaluation of the cytokine profiles in breastfed infants, 2′FL supplemented formula fed infants, and standard dairy-based formula fed infants demonstrated that 2′FL supplemented formula fed infants had lower plasma concentrations of IL-1α, IL-1β, IL-6, TNF-α, and IL-1rα than the standard formula fed infants, and were similar to those that were breastfed (155). 2′FL supplemented formulas have been approved and are being marketed in Europe (156) and the US, however, the supplementation is at much lower concentrations of 2′FL than what is found in human milk. Sialic acid concentrations have also been evaluated in human milk from mothers with term and preterm infants and compared to several infant formulas (157). The highest concentration was noted in colostrum and then decreased over the next 3 months. Milk from mothers with preterm infants had higher levels of sialic acid. Formulas, however, had a much lower sialic acid content, <25% of what was found in human milk (157). Sialic acid is integral to neonatal brain development and childhood malnutrition, specifically decreased sialic acid intake, has been linked to persistent cognitive deficits (158, 159). Thus, future studies of formulas supplemented with sialic acid would need to be tested for the cognitive function in infants and HMO supplementation to formula is an avenue to pursue in the near future.

## Human Milk Microbiota Impacts Colonization of Gut Microbiota and Likely Immune System During Neonatal Period

Different maternal factors including pathologies of the breast, intrapartum antibiotics, maternal health, body mass index (BMI), parity, gestational age, and geographic location of the mothers can contribute to shaping the milk microbiota (160–166). The early establishment of infant microbiota relies on maternal microbiota and plays a key role in the formation of the gut barrier and the maturation of the immune system (Figure 1C) (167). Human milk contains a complex community of bacteria (161, 168) which includes, but is not limited to, multiple genera from *Bifidobacterium and Lactobacillus* spp, *Streptococcus, Staphylococcus, Ralstonia, Bacteroides, Enterobacter*, and *Enterococcus* (161, 167, 169–171). Hunt et al. (172) showed that while there are common genera found in milk, there is variation overtime and between mothers. While most studies have focused on human milk bacterial content, several recent studies have noted fungi present in human milk (173–177). These studies are observational and further investigation is required to evaluate fungal population variance between mothers, the functions of milk mycobiome in infant gut development, and its interactions with other milk microbiota/bioactives and infant immune system. Due to this constraint, this review will focus on human milk and infant microbiota.

Human milk microbiota likely establishes a healthy profile of intestinal bacteria, leading to the maturation of the innate and adaptive immune systems in infants. For instance, intestinal bacteria promote the development of B-cells in Peyer's Patches and increase the release of mucosal IgA, which acts as the first line of defense (178, 179). Human milk bacteria can also improve the activity against infections through the induction of cytotoxic Th1 cells maturation *in vitro* (180). Interestingly, *Lactobacillus* in the human milk may enhance the release of Th1 cytokines and TNF-α, and activate NK cells, CD4^+^, and CD8^+^ T-cells and regulatory T-cells (181). In addition, commensal bacterial in human milk such as *Lactobacillus gasseri* and *Lactobacillus crispatus* have adhesion capacity to the intestinal cells, indicating greater colonization for beneficial bacteria in the gut in breastfed infants (182). In a recent study, Damaceno et al. (182) reported that *Bifidobacterium breve, Lactobacillus gasseri* and *Streptococcus salivarius*, limit pathogen adhesion to intestinal epithelial cells *ex vivo* (182). The microbial species identified in human milk have pathogen inhibition and improving immune function properties. Many studies compare human milk bacterial content to stool content of infants. Human milk microbiota composition is also dependent on pumped vs. directly breast fed. Recently, Moossavi et al. (161) noted that providing pumped milk was associated with higher levels of potential pathogens (i.e., *Enterobacteriaceae* and *Enterococcaceae*). Infants fed pumped milk had a lower amount of *Bifidobacterium* in their stool. In addition, Fehr et al. (183) noted that exclusively breastfed infants have a different microbiome than those that are fed pumped milk. The fact that direct breastfeeding vs. pumped milk feeding results in a different gut microbiome in infants needs to be investigated further. It is possible that some of the variations are due to variability in pump hygiene, mothers skin microbiota, and contribution from environment.

Commensal bacteria in human milk may play protective roles against gastrointestinal infections during infancy. Malago et al. (184) found that *Lactobacillus casei, Lactococcus lactis* and *Bifidobacterium infantis* suppressed the release of IL-8 in Caco-2 intestinal cell line incubated with pathogenic *Salmonella*, supporting the notion that human milk bacteria could protect the infant intestine against epithelial damage. In a recent study, higher abundance of *Bifidobacterium* at 1 week of life was associated with higher levels of IL-13, IL-5, IL-6, TNF, and IL-1β at 36 months of age compared to children with lower abundance of *Bifidobacterium* at the same time point (185). *Bacteroides* might also play a key role to support the immune system in infants during the early stages of life. In particular, the surface of *Bacteroides fragilis* has polysaccharide A which increases FOXP3 T-cells in the lamina propria resulting in suppression of inflammation (186). In a mouse model, Donaldson et al. (187) showed that *Bacteroides* binds IgA which allows it to colonize the gastrointestinal tract. In conclusion, milk microbiota likely is one of the first things to colonize the infant gut, promote growth of beneficial microbiota, and in turn impact the immune system in infants.

The infant diet also impacts the microbiome of the gastrointestinal tract and immune system in both animal models and clinical studies. In a rhesus macaques model, formula fed infants were noted to have a different gut microbiome including more *Ruminococcus* and less *Lactobacillus*. They also had an increase in pro-inflammatory cytokines TNFα, IFN-γ, IL-1β, and IL-8 (as well as several others) at 1 month of life that decreased overtime (188). Mothers milk fed rhesus macaques are noted to have more memory T-cells as well as T-helper 17 cells compared to formula fed which persists even 6 months after weaning (189). A study of juvenile rhesus macaques noted continued differences, in particular, higher CD8^+^ T-cell activation (190). These studies show that in rhesus macaques, mothers milk improves immune response while formula changes the microbiome and increases inflammation. There are also several studies carried out with a piglet model that explore diet and its effect on microbiome and the immune system. While many piglet models use sow-fed piglets, this leads to confounding factors due to housing environment, sow milk microbiota, and the maternal environment. Studies from our team housed piglets in the vivarium and fed a regulated diet to eliminate the confounding factors associated with a sow-fed piglet model. Piglets were fed either donor human milk or formula and monitored closely for growth and immune responses. Those fed human milk had a stronger immune response to vaccination in comparison to those fed formula. The piglets who received human milk had lower genera diversity at day 50. At day 21, those fed human milk had higher levels of *Bacteroides* than those fed formula (191, 192). The human milk fed group also had higher levels of T-cell proliferation (191, 192). These results were similar in comparison to infants fed human milk suggesting the strength of the model. For example, in a small comparative study, fecal samples were collected during the first 20 days of life from 6 breastfed and 6 formula fed infants. In breastfed infants, *Bifidobacterium* became the most common gut bacteria while in formula fed infants, *Bacteroides* and *Bifidobacterium* were found in similar amounts (193). Several other studies have found that in early life, stool *Bifidobacterium* amount varies in healthy breastfed infants (194–197). Although the reason is unclear, environment may play a role in this. A recent study found three distinct infant gut microbiota, one low in *Bifidobacterium* but with higher amounts of *Streptococcus*, one with high amounts of both *Bifidobacterium* and *Bacteroides*, and one with higher amounts of *Bifidobacterium*. Overtime, infant stool transitioned from the profile low in *Bifidobacterium* to a profiler higher in *Bifidobacterium* (197). The CHILD cohort has published several studies on infant diet and its impact on microbiome. At 3 months of age, formula fed infants had higher richness and increased *Lachnospiraceae*. Infants who were breastfed but briefly supplemented with formula had lower levels of *Bifidobacteriaceae* and higher levels of *Enterobacteriaceae* at 3 months of age compared to those who did not receive any formula (198). A smaller subset from this cohort noted that formula fed infants had increased richness at 4 months and higher amounts of *Clostridium difficile* were noted (195). A 2-year study of infant diet and microbiome revealed that formula feeding in the first 3 months of life is associated with decreased diversity and richness at 12–24 months of life. It is also associated with altered beta diversity (199). Andersson et al. (200) compared infants fed 3 different types of formula to breastfed infants and evaluated immune response through 6 months of age. The breastfed group had an increase in leukocyte count, particularly an increase in neutrophils. Formula fed infants had a decrease in the relative amount of NK cells and an increase in CD4^+^ αβT-cells. Formula fed infants also had a higher ratio of CD4–CD8 cells (200). Data from these studies indicate that human milk feeding is optimal for microbial colonization, promoting robust immune response and decreasing inflammation in early life.

## Extracellular Vesicles and Microrna Cargo Role in Immune Function

Extracellular vesicles is a broad term used to describe vesicles released from many cell types. Readers are referred to O'Reilly et al. (201). for a detailed review of human milk extracellular vesicles (EVs) and their role on infant health. The different methodologies (ultracentrifugation, Exoquick) used to isolate EVs indicate the existence of two subsets such as exosomes (30–100 nm) (202–204) and microvesicles (100–1,000 nm) (205, 206). EVs have been reported to contain various molecules (i.e., proteins, microRNA, metabolites) (207–215). It is yet to be determined whether both exosomes and microvesicles contain miRNAs as most of the methods used so far enrich exosomes. Interestingly, milk seems to contain the highest level of miRNAs compared to its volume. The mechanisms involved in loading the miRNAs to EVs in human milk are still unclear and future research is needed. For a more detailed review of EV biogenesis and cargo composition readers are referred to Spencer and Yeruva (216). The focus of this subsection is to describe EV-microRNA cargo role on infant health.

miRNA are small non-coding RNA (~22 nucleotides) that regulate post-transcriptional expression of genes and have biological activities in humans (217–219). Human milk contains several miRNAs (218, 220), and these miRNAs survive in the acidic environment in the GI tract and can be absorbed (221). Infant formulas, however, have a significantly lower amount of miRNAs compared with human milk (218, 222). The origin of these miRNAs is still under debate. However, based on the current knowledge on the composition of the EV proteins, breast cell lines, and miRNA profile of mammary gland cells, these miRNAs are likely from immune-related and mammary gland cells (223–225). The literature review of several studies on miRNA profile suggests that miR-148a-3p, miR-22-3p, miR-200a-3p, miR-146b-5p, miR-30d-5p, let-7a-5p, miR-30a-5p, let-7f-5p, let-7b-5p, and miR-21-5p (226–231) were the most abundant in human milk. *In vitro* studies suggest that milk miRNAs are taken up by intestinal, immune, and cancer cell lines (218, 220, 232–236). Future animal models and clinical studies under controlled conditions are needed to determine the bioavailability of these miRNAs.

Few studies have been conducted so far on various factors impacting milk miRNA composition. For example, in mice fed high-fat diet, changes in milk miRNA expression was observed (237). Target prediction analysis of these miRNAs in the high-fat diet group impacted developmental process and transcription. Most recently, Carney et al. demonstrated changes in miRNA profile based on delivery status (preterm vs. term) that appear to influence metabolism and lipid biosynthesis. This suggests gestational age likely plays a role in milk miRNA composition and miRNAs appear to directly influence neonatal health and metabolism. This is an area for future studies to determine the underlying mechanisms involved in milk miRNA composition.

The biological impact of human milk EV-miRNAs on infant health is important to address before supplementing formulas. Previous studies using target prediction analysis of human milk miRNAs provided initial evidence that the majority of these miRNAs are likely impacting the immune system. Also, experimental evidence from *in vitro* and *in vivo* studies using infection and inflammation models suggest that milk miRNAs could impact the immune system. For example, miR-148, present in pre-term and term human milk but significantly lower in formula (218, 226), appears to be the most abundant in human milk. It is shown to regulate the innate immune response in several ways including limiting cytokine production (238). miR-148 also inhibits T-cell proliferation initiated by the presentation of antigens by dendritic cells in a mouse model (238). Let-7 functions to regulate the innate immune system; it limits B-cell activation, affects T-cell differentiation, and regulates TLR4 signaling and macrophage activation (239, 240). miR-30 is important for intestinal epithelial cell homeostasis (241) and the immune response to *Mycobacterium tuberculosis* (242) and influenza infections (243). miR-30 also inhibits antigen processing and presentation by dendritic cells and macrophages (244). Other studies identified miR-181 in human milk (220) which induces B- and T-cell differentiation and development (245, 246) and plays a role in inflammation by downregulating TNF-α production in *Brucella abortus* infections (247). In addition, porcine milk miRNAs were recently shown to reduce LPS-induced apoptosis by preventing TLR4 in intestinal epithelial cells (248). Thus, it is possible that milk miRNAs protect infants from infection, reduces inflammation, and boosts the immune response by various mechanisms (Figure 1D).

The potential for human milk miRNAs acting as TLR7 ligand is a novel concept that we put forth in this review. We hypothesize that GU rich motif (GU or GUUG) of human milk miRNAs activates TLR7/TLR8 and could have an adjuvant effect on immune response during vaccination in breastfed infants. For example, milk miR-21, let-7a, and let-7b have a GU rich region and can bind to TLR7/TLR8 receptors (249–252). Thus, milk miRNAs could have dual functions such as TLR7/TLR8 receptors and/or regulatory role by inhibiting gene expression. Mechanistic studies are needed to determine the specific role of milk miRNAs. In addition, whether miRNAs have direct or indirect effects via microbiota on the infant gut and the immune system is not fully understood. However, the evidence so far suggests that miRNAs could change microbiota composition. Recently, exosome/RNA depleted diet (based on bovine milk exosomes) fed C57Bl6 mice showed changes in the composition of microbiota with relative abundances reported < 1% at family taxonomic level in comparison to exosome/RNA sufficient diet fed mice (253). This study does not show the direct role of miRNAs from bovine milk, nor does it indicate which components of exosomes altered the microbiota composition. However, in a different study it has been demonstrated that bacterial growth is promoted in the presence of certain miRNAs and that endogenous miRNA produced by intestinal epithelial cells alter gut microbial diversity. The increased growth was observed in co-culture of Mission® miRNA mimics and *Fusobacterium nucleatum* (ATCC® 10953) and *E.coli* (ATCC® 47016) (254). Results from this study suggest that miRNAs modulate the gut microbiota; to date, however, no studies investigating the effect of exogenous miRNAs from human milk on neonatal microbiota have been conducted. If miRNAs do indeed promote the survival and growth of gut bacteria, these may serve as a novel component to supplement the infant diet.

## Perspective and Conclusions

Human milk remains the gold standard for infant nutrition. This review summarized several bioactive components of human milk and their impact on infant microbiome and gut/immune function. Human milk oligosaccharides have been shown to have a prebiotic effect, decrease infectivity as pathogen decoys, and enhance the immune system. Milk microbiota appears to help infants' gut and immune system and protect from pathogens. However, several questions remain unanswered that could ultimately improve term and preterm infant outcomes including decreased infection and improved gut and immune function. Mechanistic studies involving animal models in association with clinical trials are needed. While large animal models (piglet and monkey) are advantageous due to the similarities with infant gut physiology (189, 255), they have multiple limitations. These include a low cost-benefit ratio to generate germ-free animal models due to the specialized facilities required, difficulty and expense of knock-out models, issues obtaining species specific reagents and ethical constraints. Animal models have shown differences in offspring gut microbiome and immune response based on diet. Clinical data, while extremely relevant, only allows for association data due to confounding factors. Thus, alternative models such as germ-free mice could be explored to understand the mechanistic questions about milk bioactives. Determining how different human milk bioactives individually and in combination will impact infants' health needs to be pursued.

### Future Research

While many questions relating to human milk bioactives have been addressed, there are areas of research that requires future studies. The questions that remain unanswered are: (1) what combination of HMOs or their derivatives should be added to standard formula? (2) should HMOs be added to formula for premature infants? (3) what are the direct and indirect effects of HMOs on infant immune function? (4) how does maternal microbiota transfer into milk and further shape the milk microbiome? (5) does out-of-body bacteria, including skin bacteria, infant oral bacteria, or bacteria from the environment enter the mammary gland and alter milk microbiota? (6) does milk microbiome affect composition of other milk components such as HMOs and miRNAs? (7) how does milk microbiota affect TLRs in the infant gut and does this impact colonization with commensal bacteria and protection from invading pathogens? (8) does the gut milieu (microbiota and mycobiota) interact and how does the interplay impact overall infant health? and (9) how does the addition of different human milk components to formula impact the gut colonization patterns, and in turn, longitudinal infant health? All these questions need further investigation using preclinical and clinical studies. microRNAs are a newer field of study, thus, many questions remain pertaining to how miRNAs interact with the infant gut microbiome and immune system. In conclusion, determining how different human milk bioactives individually and in combination will promote infants' health needs to be pursued.

## Author Contributions

LC, AE, FR, MV, and LY conceived and wrote the paper. FR made the figures. DM and KM edited the manuscript. All authors contributed to manuscript revision and read and approved the submitted version.

## Conflict of Interest

The authors declare that the research was conducted in the absence of any commercial or financial relationships that could be construed as a potential conflict of interest.
